# Harmony in Motion: Unraveling the Nexus of Sports, Plant-Based Nutrition, and Antioxidants for Peak Performance

**DOI:** 10.3390/antiox13040437

**Published:** 2024-04-04

**Authors:** Asma Ayaz, Wajid Zaman, Zsolt Radák, Yaodong Gu

**Affiliations:** 1 Faculty of Sports Science, Ningbo University, Ningbo 315211, China; asma@nbu.edu.cn; 2 Department of Life Sciences, Yeungnam University, Gyeongsan 38541, Republic of Korea; wajidzaman@yu.ac.kr; 3 Research Institute of Sport Science, University of Physical Education, 1123 Budapest, Hungary; radak@tf.hu; 4 Faculty of Sport Sciences, Waseda University, Tokorozawa 359-1192, Japan

**Keywords:** sports, plant-based nutrition, antioxidants, oxidative stress, athletic performance

## Abstract

The intricate interplay between plant-based nutrition, antioxidants, and their impact on athletic performance forms the cornerstone of this comprehensive review. Emphasizing the pivotal importance of dietary choices in the realm of sports, this paper sets the stage for an in-depth exploration of how stress and physical performance are interconnected through the lens of nutrition. The increasing interest among athletes in plant-based diets presents an opportunity with benefits for health, performance, and recovery. It is essential to investigate the connection between sports, plants, and antioxidants. Highlighting the impact of nutrition on recovery and well-being, this review emphasizes how antioxidants can help mitigate oxidative stress. Furthermore, it discusses the growing popularity of plant-based diets among athletes. It elaborates on the importance of antioxidants in combating radicals addressing stress levels while promoting cellular health. By identifying rich foods, it emphasizes the role of a balanced diet in ensuring sufficient intake of these beneficial compounds. Examining stress within the context of sports activities, this review provides insights into its mechanisms and its impact on athletic performance as well as recovery processes. This study explores the impact of plant-based diets on athletes including their types, potential advantages and challenges. It also addresses the drawbacks of relying on plant-based diets, concerns related to antioxidant supplementation and identifies areas where further research is needed. Furthermore, the review suggests directions for research and potential innovations in sports nutrition. Ultimately it brings together the aspects of sports, plant-based nutrition, and antioxidants to provide a perspective for athletes, researchers and practitioners. By consolidating existing knowledge, it offers insights that can pave the way for advancements in the ever-evolving field of sports nutrition.

## 1. Introduction

### 1.1. Background

Athletic performance is closely connected to a variety of factors, with nutrition being a crucial driver [1]. The importance of food choices in optimizing training regimens and improving recovery has become a prominent focus in sports science [2]. From infancy through adolescence to adulthood, lifestyle factors such as nutrition, exercise, and other habits play a pivotal role in shaping athletic abilities and overall health. Vandoni et al. suggested that optimal nutrition and lifestyle habits during early developmental stages can have long-lasting effects on sports performance and health outcomes later in life [3]. Factors such as breastfeeding duration, introduction to solid foods, and physical activity levels during childhood can influence growth, development, and athletic potential. During adolescence, a critical period marked by rapid growth and development, nutritional intake becomes even more crucial for supporting physical activity and optimizing performance. Adolescents engaging in sports activities may have increased energy and nutrient requirements, necessitating a balanced diet rich in essential nutrients to fuel growth, support muscle development, and enhance recovery [4,5]. Nutrition has the dual purpose of providing energy to the body and maintaining physiological equilibrium. It also plays a vital function in supporting the metabolism of energy and facilitating the body’s response to exercise-induced stress [6]. Athletes face the challenge of managing oxidative stress, a natural byproduct of cellular metabolism and physical exertion while pursuing peak performance [7]. Oxidative stress arises when there is an imbalance between the production of reactive oxygen species (ROS) and the body’s ability to counteract them with antioxidants [8,9]. While moderate levels of oxidative stress are integral to training adaptations, severe or sustained oxidative stress can cause cellular damage, and inflammation, and hinder recovery [10].

The complex interplay between oxidative stress and antioxidant defense mechanisms highlights the intricate connection between exercise and nutrition [11,12]. Athletes and researchers are acknowledging the growing importance of optimizing nutritional techniques to regulate oxidative stress, which can lead to enhanced performance and reduced risk of overtraining-related issues [13]. An observable pattern in modern sports nutrition is the increasing fascination with diets that mostly consist of plant-based foods [14,15]. Plant-based diets are becoming increasingly popular among athletes due to their potential to supply essential nutrients, enhance recovery, and include a wide range of bioactive compounds [16,17]. These bioactive substances comprise a range of phytochemicals, such as flavonoids, phenolic compounds, carotenoids, and other phytochemicals possessing various properties and are present in fruits, vegetables, nuts, seeds, and whole grains. For example, flavonoids are recognized for their anti-inflammatory and anti-oxidant properties, while phenolic compounds support immunological and cardiovascular health [18,19]. Carotene and lycopene as carotenoids are vital for eye health and may prevent chronic illnesses [20]. Cruciferous vegetable phytochemicals like glucosinolates may prevent cancer [21]. The attractiveness of plant-based diets extends beyond nutritional criteria, embracing ethical, environmental, and performance-related aspects [22,23]. Diets of athletes that include a variety of bioactive compounds sourced from plant-based foods may enhance athletic performance and provide synergistic health benefits [24]. The study aims to offer practical insights for athletes, coaches, and nutrition professionals as they navigate the complex interaction between sports, nutrition, and overall well-being. This is achieved by a detailed examination of the existing literature.

The quest for athletic excellence involves the crucial convergence of training, nutrition, and recovery [25,26]. This article provides guidance on achieving optimal performance by exploring the intricate relationship between nutrition and sports, the delicate balance with oxidative stress, and the changing trends of plant-based diets among athletes. In the field of athletics, where achievements are frequently evaluated in terms of milliseconds and millimeters, the human body encounters exceptional physical requirements [27,28]. In this review, it becomes clear that nutrition goes beyond just providing sustenance—it has a powerful influence on energy levels, recovery processes, and the body’s ability to adapt to the demands of training.

Oxidative stress, a complex and significant factor, that is typically overlooked in the context of athletics, plays a crucial role in performance [29]. This article explores the complex physiological processes involved in the interaction between exercise intensity and reactive oxygen species, shedding light on how they influence an athlete’s progress. Oxidative stress functions as a signaling mechanism for adaptation and poses a challenge to maintaining performance over time [30]. Amidst this evolving narrative, there is a noticeable change in the dietary preferences of athletes, as they increasingly lean towards plant-based nutrition [15]. This nutritional paradigm goes beyond health considerations and can redefine performance boundaries [31]. The reasons driving athletes to choose plant-based diets are varied, demonstrating a symbiotic relationship between plant-based nutrition and the quest for peak athletic performance [32]. This article serves as a comprehensive reference for athletes, coaches, and nutrition enthusiasts who are exploring the complex fields of nutrition, oxidative stress, and the growing popularity of plant-based diets. It seeks to provide valuable insights and information to help navigate the always-changing world of sports science. The review aims to simplify the core principles of nutritional strategies, understand the complexities of oxidative stress management, and highlight the way to utilize the natural advantages of plant-based nutrition.

### 1.2. Objectives and Scope of the Study

This review’s main objective is to thoroughly investigate the complex relationship that exists between sports, plants, and antioxidants in order to better understand the complex dynamics that exist between these interconnected components. This involves a thorough examination of several aspects, such as the physiological impacts of sports on oxidative stress and potential functions of antioxidants in reducing cellular damage. By summarizing the goals, we aim to provide a concise overview of the study’s purpose and scope, thereby enhancing the review’s coherence and clarity.

This review goes beyond analyzing individual components and explores the synergies and interactions that occur in the human body during and after physical activity. It aims to develop a comprehensive understanding of sports’ overall impact on cellular health by investigating physiological responses to exercise-induced stress and the associated need for antioxidant defenses. Furthermore, the study investigates various types of antioxidants, with a focus on those derived from plants. This involves a thorough examination of the wide spectrum of phytochemicals present in various plants, revealing their potential benefits and mechanisms of action. The emphasis on plant-based diets as the primary concern demonstrates the commitment to researching alternative nutritional approaches for athletes and individuals who engage in regular physical activity.

Several individuals, including researchers, sports scientists, dietitians, players, and coaches, can seek significant relevance in the study. It aims to offer important insights into the current knowledge base while presenting an in-depth look at the potential advantages of incorporating plant-based, high-antioxidant meals into sports nutrition. Furthermore, the practical recommendations offered aim to bridge the gap between scientific comprehension and implementable tactics, making the information accessible and applicable to all athletes and fitness enthusiasts. Furthermore, driving this research is basic science questions aimed at elucidating the intricate relationships among antioxidants, plant-based diet, and sports. It seeks to investigate the potential of plant-based diets to enhance athletic performance, elucidate the role of antioxidants in reducing cellular damage, and investigate the physiological impacts of different sports activities on oxidative stress. While a plant-based diet is emphasized primarily, the study covers a wide range of activities, including endurance, strength training, and high-intensity exercises. It also explores the role of plant-based macro- and micronutrients, as well as the diverse array of antioxidants found in plants, underscoring the importance of phytochemicals in enhancing overall health.

## 2. Antioxidants: An Overview

### 2.1. Definition and Types of Antioxidants

Antioxidants play a crucial role in sports and nutrition by protecting against oxidative stress caused by intense physical activity [33]. Antioxidants are chemical substances that combat oxidative stress, a condition that can damage cells. They play a crucial role in maintaining cellular resilience [34,35]. Their crucial function is to counteract free radicals, which are extremely reactive molecules that, if not controlled, can initiate a series of harmful effects on cells [36,37]. As athletes strive to exceed their physical capabilities through intense workouts, understanding the significance of antioxidants becomes crucial [38,39]. These compounds function as molecular barriers, reducing the potential damage caused by free radicals and preserving the cells integrity [40,41]. Various physiological processes can be disrupted and oxidative stress, which is characterized by an imbalance between the body’s capacity to neutralize free radicals and their production, can result in cellular injury. Moreover, it contributes to the development of chronic diseases. Cellular components, including DNA, lipids, and proteins, are susceptible to damage from free radicals, which can result in structural and functional impairments that undermine the health and functionality of the cell [42,43]. Therefore, antioxidants provide an important role in maintaining cellular resilience and improving overall health, especially in individuals involved in strenuous exercise such as sports. To comprehend the complexities of oxidative protection in the realm of sports and nutrition, it is crucial to have a clear understanding of the primary categories of antioxidants (Table 1) [44].

#### 2.1.1. Vitamin C (Ascorbic Acid)

Source: Citrus fruits (such as oranges and lemons), strawberries, kiwi, and bell peppers are rich in this nutrient [56,57].

Role: Vitamin C stands as a versatile water-soluble antioxidant [58]. In addition to its function in neutralizing free radicals, it plays a crucial role in the production of collagen, which is essential for preserving the structural integrity of the skin, connective tissues, and blood vessels [59,60]. Its immunomodulatory characteristics render it essential for athletes experiencing physical stress [61,62].

#### 2.1.2. Vitamin E (Tocopherols and Tocotrienols)

Source: Present in nuts (almonds and sunflower seeds), as well as in vegetable oils (sunflower oil and olive oil, etc.) [63,64].

Role: Lipid-soluble compounds, tocopherols and tocotrienols are present in cell membranes, protecting them against oxidative damage [65]. Vitamin E enhances the overall cellular health of athletes, strengthening immunological function and aiding in the recovery process after strenuous workouts [66,67].

#### 2.1.3. Beta-Carotene

Source: Beta-carotene is found in large quantities in orange and yellow vegetables such as carrots and sweet potatoes, as well as in leafy greens like spinach and kale, etc. [68,69].

Role: Beta-carotene, a vivid pigment, acts as a precursor to vitamin A—an essential nutrient for vision, immunological function, and skin health [70,71]. In addition to its function as a potent antioxidant, beta-carotene enhances the body’s defenses against oxidative stress, making it a valuable component in the nutritional regimen of athletes [72,73].

#### 2.1.4. Polyphenols

Sources: Polyphenols are present in a wide range of plant-based foods, such as fruits (apples, berries), vegetables (onions, broccoli), tea, and red wine [74,75].

Role: Polyphenols, a wide range of antioxidants, contribute to the vivid hues of fruits and vegetables [76,77]. This classification includes flavonoids, phenolic acids, and other subcategories, each providing distinct advantages for one’s health [78,79]. The antioxidant, anti-inflammatory, and antibacterial properties of polyphenols help athletes maintain good cardiovascular health and general wellness [67,80].

#### 2.1.5. Selenium

Sources: Nuts (especially Brazil nuts), seeds, seafood, and whole grains are the most common foods to acquire selenium [81,82].

Role: Glutathione peroxidase and other antioxidant enzymes rely on the trace element selenium for proper activity [83,84]. Athletes receive benefit from selenium because of its function in balancing oxidative processes by counteracting reactive oxygen species [85,86].

Athletes, coaches, and nutritionists can use this in-depth look into important antioxidants as a starting point for making dietary selections that support optimal cellular health and performance. A better grasp of nature’s intricate defense mechanisms is made possible by the synergistic effects of these antioxidants, which aid athletes in their pursuit of peak performance.

### 2.2. Importance of Antioxidants in Human Health

Often disregarded despite their critical importance, antioxidants play a pivotal role in human health [87]. They are crucial to cellular health, resilience, and disease prevention, and their significance extends far beyond basic biochemical events [88,89]. The maintenance of the intricate equilibrium between oxidative stress and antioxidant defense mechanisms within the human body is heavily reliant on the presence of antioxidants [90]. Oxidative stress arises from a disparity between the production of reactive oxygen species (ROS) and the body’s ability to counteract them, which can cause damage to cells, inflammation, and the development of various diseases [8]. Antioxidants serve as protectors, counteracting harmful free radicals and protecting cells against oxidative damage. Cellular resilience is augmented through the facilitation of repair mechanisms, modulation of signaling pathways implicated in inflammation and apoptosis, and preservation of cellular structural integrity [91]. As we delve deeper into their importance, we see that antioxidants are more than just dietary components; they are guardians that navigate the intricate paths of human health [92,93]. Antioxidants promote overall wellness by assisting the body’s capacity to adjust to stimuli and maintain functioning at its optimum level [94]. The nuanced game begins with the body’s natural metabolic processes, which lead to the ongoing production of free radicals. Antioxidants are important because they actively seek for and neutralize free radicals, stopping the potential impact caused by oxidative stress [95]. To keep the fine equilibrium needed for optimal cellular activity, antioxidants function as molecular barriers by destroying free radicals [96,97]. Ensuring the safety of biomolecules and cellular structures is the duty of a sentinel during this process, which goes beyond a simple biochemical transaction.

The narrative gets trickier as we learn about the link between oxidative stress and chronic health conditions. One of the main causes of many chronic diseases is oxidative stress, which occurs when the body’s ability to produce free radicals is not proportional to its capacity to neutralize them [34,98,99]. Heart disease, neurological diseases, and even some cancers have this behavior as their root cause [100,101]. By disrupting this delicate equilibrium, antioxidants offer protection against the progressive worsening of chronic diseases [102]. We highlight the antioxidants’ inherent preventive capacity by investigating this connection. When it comes to cellular health—the basis of overall wellness—antioxidants play a pivotal role [103,104]. Beyond its ability to neutralize free radicals, antioxidants exert a wider range of impacts. The intricate regulation of gene expression [105], cellular communication [106], and DNA repair mechanisms [107], all include their active participation. In addition to coordinating and improving the functioning of cellular processes, their capacity to reduce oxidative stress is a key component of their multifaceted function. In this context, the absence of disease is secondary to the vitality and robustness of individual cells.

### 2.3. Dietary Sources of Antioxidants

The nutritional aspect plays a crucial role in supporting human health through antioxidants, serving as a fundamental element [108]. The aim is to explore and understand the diverse array of foods that are abundant sources of these essential compounds. The most powerful manifestation of antioxidants is not limited to laboratory formulations, but rather can be found in a wide variety of fruits, vegetables, nuts, and seeds that are often consumed [109,110]. By examining these food sources, we discover not only a compilation of essential nutrients, but also a harmonious blend of culinary elements that promote well health.

**Figure 1:**
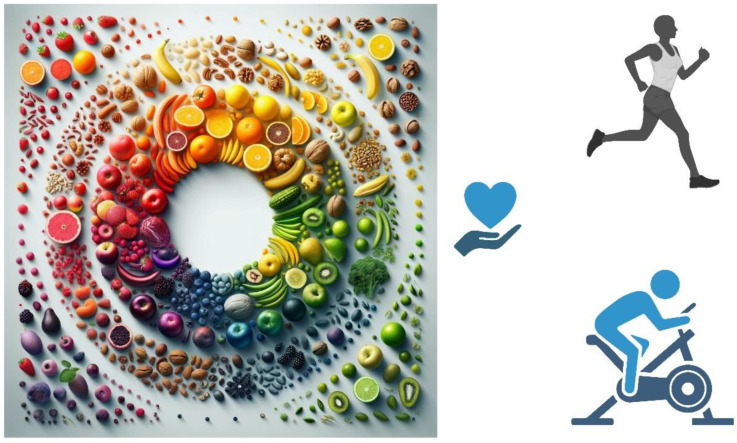
Comprehensive overview of antioxidant-rich sources in plant-based foods. This figure was created with BioRender.com (accessed on 28 February 2024).

As we begin this exploration, it is crucial to acknowledge that antioxidants are not difficult to find and are not limited to specialized superfoods. They are present in the regular foods that make up a balanced diet [111]. Fruits, with their vivid colors, are true repositories [112], berries, including blueberries, strawberries, and raspberries, are notable not only for their pleasant flavor but also for their significant amounts of anthocyanins, a powerful group of antioxidants recognized for their ability to protect against oxidative stress (Figure 1) [113,114]. Vegetables, which are essential for a diet rich in nutrients, make a substantial contribution to the collection of antioxidants [115]. Dark, leafy greens such as spinach and kale contain significant amounts of vitamins A, C, and E, making them powerful friends in the fight against free radicals [116]. Moreover, cruciferous vegetables such as broccoli and brussels sprouts contain a significant amount of sulforaphane, a chemical renowned for its antioxidant and anti-inflammatory properties [117,118].

Nuts and seeds, renowned for their nutritious compositions, enhance the narrative surrounding antioxidants [119]. Almonds, walnuts, and sunflower seeds are rich in vitamin E, while brazil nuts are notable for their selenium concentration, which is an essential trace mineral for the production of antioxidant enzymes [120,121]. The array of antioxidants goes beyond the usual, demonstrating that having a diverse diet is not only enjoyable but also a deliberate way to increase antioxidant consumption [122]. By incorporating foods rich in antioxidants into our diet, it becomes clear that a balanced diet is not just a nutritional recommendation, but also an effective method to increase antioxidant levels [123]. The combination of several foods, each providing its distinct combination of antioxidants, creates a comprehensive method to guarantee sufficient consumption [124]. It emphasizes the significance of having a varied diet, encouraging consumers to explore the wide range of natural foods in order to obtain the complete range of antioxidant benefits [125].

## 3. Sports and Oxidative Stress

### 3.1. Introduction to Oxidative Stress in Sports

**Figure 2:**
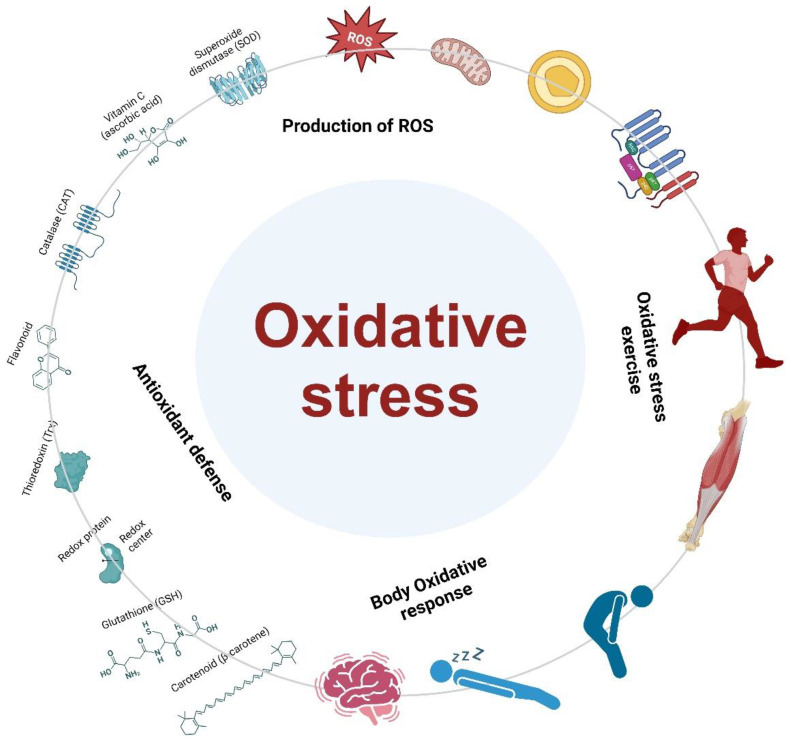
Visual overview of oxidative stress mechanisms and biochemical pathways during physical activity in sport. This figure was created with BioRender.com (accessed on 6 February 2024).

Within the field of sports science, the dynamic relationship between physical activity and oxidative stress is a captivating narrative that influences how players approach their training, recovery, and overall performance [126]. The aim is to explain the notion of oxidative stress in the context of sports, which acts as a physiological stimulus for adaptation and a possible indicator of injury. In this investigation, we examine the complexities of oxidative stress, analyzing its precise meaning, comprehending its occurrence during physical activity, and acknowledging its ambivalent role as both an ally and adversary in the realm of sports. The core of this investigation is around the understanding that oxidative stress is not a detrimental outcome of physical exertion, but rather a fundamental physiological response that is essential to the adaptive processes triggered by sports training [127]. Oxidative stress, in the context of athletics, refers to an imbalance between the generation of reactive oxygen species (ROS) and the body’s capacity to counteract them via antioxidant defenses (Figure 2) [128,129]. The delicate balance is greatly affected by the intensity, duration, and nature of physical activity, which is essential knowledge for both athletes and sports professionals [86,130].

Oxidative stress occurs in sports when the formation of reactive oxygen species (ROS) exceeds the body’s ability to counteract these molecules, potentially causing damage to cellular structures [130,131]. During vigorous physical exercise, athletes experience an elevation in oxygen consumption, which promotes the production of reactive oxygen species (ROS) [132,133]. The increased level of oxidative stress becomes especially apparent during activities that require substantial aerobic metabolism, underscoring the significance of identifying the precise circumstances in which athletes may be more vulnerable to oxidative stress [134,135].

Sports and exercise are inherently characterized by physical exertion, which serves as a stimulant for the production of ROS [128,136]. The generation of free radicals is a result of muscular contractions, heightened oxygen consumption, and metabolic activities that occur during exercise [137]. Although it may be initially seen as a potential threat, it is crucial to emphasize the dual function of oxidative stress in the field of athletics, which goes beyond its reputation as a simple adversary. Oxidative stress, when examined with a sophisticated perspective, unveils its complex and diverse characteristics. It serves as a signaling system that prompts adaptive responses in the body, leading to enhancements in endurance, strength, and overall performance [138]. However, if the body’s ability to adapt is exceeded by oxidative stress, it can become a possible cause of damage. This can lead to muscle fatigue, inflammation, and in the long run, it may compromise sports well-being [139,140]. Achieving this delicate equilibrium requires a combination of skill and knowledge for athletes and their support teams, emphasizing the importance of customized plans that utilize the beneficial effects of oxidative stress while minimizing its potential disadvantages.

### 3.2. Mechanisms of Oxidative Stress during Physical Activity

The physiological aspects of sports are greatly affected by oxidative stress, a process that happens during physical exertion [141]. Oxidative stress during exercise is not just a common side effect of exercise, it is a potent factor that determines the adaptive responses in the human body (Figure 2). This inquiry aims to analyze the complex biological mechanisms that generate it [142]. To better comprehend the molecular mechanisms, it is crucial to grasp the origin of oxidative stress, which is closely linked to the generation of reactive oxygen species (ROS) [143,144]. An elevated oxygen demand is associated with physical activity, especially that which involves an increase in aerobic metabolism [145,146]. There are pros and downsides to increasing oxygen consumption, as it results in more reactive oxygen species (ROS) being produced [147,148]. These highly reactive molecules, including free radicals such as superoxide anion and hydroxyl radical, are produced naturally as byproducts of cellular respiration and metabolic activities during physical exercise [149].

The effect of oxidative stress varies significantly and is intimately influenced by the three key factors of exercise: intensity, duration, and type [150]. High-intensity activities, which are known for their demanding requirements on energy systems, frequently result in an increased rate of reactive oxygen species (ROS) formation [151]. Engaging in long periods of physical activity, particularly in endurance sports, increases the amount of time that the body is exposed to oxidative stress, which might potentially amplify its impact [152]. In addition, the specific type of exercise, whether it is aerobic or anaerobic, creates unique metabolic requirements that affect the type and degree of oxidative stress that athletes encounter [153,154].

Mitochondria, commonly recognized as the primary source of energy in cells, are revealed to play a crucial role in the story of oxidative stress [155,156]. Although they function as the principal locations for the formation of reactive oxygen species (ROS), they are also equipped with complex defense mechanisms, including antioxidant enzymes, to ensure cellular balance [157,158]. The intricate equilibrium between the production of reactive oxygen species (ROS) in mitochondria and the protective mechanism against oxidative damage plays a crucial role in influencing the adaptive responses triggered by oxidative stress during physical exercise [159,160]. A potent combination of oxidative stress and inflammation, a normal response to tissue damage or stress, is created in the context of exercise-induced physiological changes [135,161]. Chronic inflammation due to persistent oxidative stress poses risks for athletes, even while minor inflammation is thought of as a typical aspect of the body’s adaptive response [10,162]. The complex mechanisms that control the body’s response to exercise can be better understood by delving into the relationship between oxidative stress and inflammation [163,164].

### 3.3. Impact of Oxidative Stress on Athletic Performance

Athletes’ abilities are greatly affected by an association between exercise and oxidative stress [165]. Starting with the biological foundations and progressing to the tangible outcomes that influence the sports world, this discussion aims to explore the multiple ways in which oxidative stress impacts an athlete’s performance (Figure 2). Athletes’ performance is significantly impacted by oxidative stress, which is a normal response to intense physical exertion [161,163,166,167]. The effects of this oxidative environment on muscle fatigue and recovery are especially noticeable [168]. Athletes risk muscular fatigue due to the persistent generation of reactive oxygen species (ROS) in their pursuit of peak performance. This has an impact on both the short-term performance and the long-term training adaptations. In order to improve performance, strength, and endurance, athletes have to understand the delicate balancing act between oxidative stress and adaptive responses within muscle fibers [169,170].

The narrative takes on new depths when we consider the intricate relationship between oxidative stress and damage. The capacity of exercise to increase strength and resilience is well-known. But the balance could shift in favor of susceptibility if oxidative stress continues. Potential harm could be increased by oxidative damage to tissues along with the subsequent inflammatory response [171,172]. Acknowledging this potential connection is critical because it encourages athletes and their support staff to take a holistic approach that targets both performance improvement and injury prevention using innovative techniques. When considering the effects of oxidative stress on athletic performance, it becomes clear that there is a requirement for efficient methods to control this physiological reaction [173]. Recognizing that oxidative stress is an inherent component of exercise, the attention turns to utilizing its beneficial elements while minimizing potential disadvantages [174]. In order to more effectively protect themselves against oxidative stress, athletes are encouraged to follow specific dietary protocols that include consuming foods rich in antioxidants [175]. Optimizing training routines, ensuring sufficient recovery, and incorporating sophisticated rest intervals are just as important as diet. A complete strategy that helps athletes perform well under conditions of high exertion and oxidative stress must take these into account [72,162,176,177].

## 4. The Role of Plants in Sports Nutrition

### 4.1. Plant-Based Diets for Athletes

Plant-based diets have been a central focus in the constantly changing field of sports nutrition. They are not only a food preference, but a holistic lifestyle approach for athletes [178]. The aim is to explore the idea of plant-based nutrition in the context of sports, which goes beyond conventional dietary boundaries and is increasingly popular among athletes who are looking to achieve peak performance and overall well-being. The core of this investigation revolves around the introduction of plant-based diets, which refer to dietary patterns primarily focused on consuming plant-derived foods. The transition towards a plant-based diet represents a deviation from traditional sports nutrition practices, which frequently give preference to protein sources originating from animals [179]. Athletes are encouraged to adopt a new way of thinking that involves embracing the wide range of plant-based foods, such as fruits, vegetables, whole grains, legumes, nuts, and seeds [180,181,182,183].

Plant-based diets include natural adaptability, allowing for the inclusion of diverse dietary preferences and limits [183]. These variations encompass vegetarianism, which excludes animal products except for dairy and eggs, and veganism, which excludes all animal products [184]. Within this range, athletes may choose to follow a flexitarian strategy, which involves including plant-based meals while not completely eliminating animal products [179]. Appreciating this variety is crucial for comprehending the intricate methods by which athletes manage plant-based nutrition to achieve their distinct performance and dietary objectives.

The popularity of plant-based diets among athletes has experienced significant momentum [14,185,186]. In addition to ethical concerns, athletes are attracted to plant-based diets due to their alleged health advantages, environmental sustainability, and potential to improve athletic performance [187]. Athletes from many sports have enthusiastically adopted plant-based diets, highlighting the practicality and benefits of including more plant-based foods in their conventional training routines [187,188,189]. The potential benefits and drawbacks of athletes switching to plant-based diets are covered in this study. Improving cardiovascular health, speeding up recovery due to plant-based diets’ anti-inflammatory properties, and perhaps better weight control are all benefits [190,191]. Sufficient protein consumption, nutritional shortages, and managing the social and practical demands of a plant-based lifestyle with hard training can be challenging [185].

### 4.2. Nutrient-Rich Plants for Sports Nutrition

Identifying nutrient-rich plants that are necessary for a balanced diet is a major focus in the area of plant-based nutrition for athletes [186]. Researchers need to find plant-based diets that are high in essential nutrients for peak performance on the field. This aims to emphasize the need of strategic fueling that caters to the specific requirements of sports. However, understanding the nutrient composition of non-plant-based sports diets is equally essential in order to provide athletes with a complete understanding of nutritional options. This comparison reference point may facilitate informed decision-making regarding dietary choices for athletes, considering elements such as nutrient adequacy, performance optimization, and overall health outcomes (Table 2) [192]. Central to this investigation is the focus on particular nutrients acknowledged as crucial elements for athletic performance. Protein, which is essential for the repair and growth of muscles, is of utmost importance [193]. Legumes like lentils and chickpeas, nuts such as almonds and walnuts, and soy products like tofu and tempeh are important sources of protein for athletes [194,195]. This examination of protein obtained from plants not only questions traditional beliefs but also emphasizes the wide range of protein-rich choices accessible to anyone adopting a plant-based diet [196].

Iron, an essential element for the transportation and consumption of oxygen, becomes another topic of emphasis [197]. Spinach, lentils, and fortified cereals are plant-based sources of iron that can help athletes fulfill their iron requirements without depending on conventional animal-derived sources [198]. The narrative transitions from perceived constraints to the ample options that enable athletes to create meals rich in nutrients that are in line with their training objectives.

Calcium, which is crucial for maintaining strong bones and proper muscle function, is found in plant-based sources such as fortified plant milks, leafy greens like kale and bok choy, and tofu made with calcium sulfate [199,200]. This investigation not only questions the traditional story that links calcium solely with dairy, but also emphasizes the flexibility and variability of plant-based sources.

Omega-3 fatty acids, known for their ability to reduce inflammation and promote heart health, are the subject of attention [201,202]. Athletes can get appropriate omega-3 consumption without relying on fish-derived options by consuming plant-based sources such as flaxseeds, chia seeds, hemp seeds, and walnuts [202,203,204,205]. This shift in perspective emphasizes the nutritional knowledge inherent in plant-based diets and their ability to meet the complex requirements of athletes.

The main message goes beyond listing nutrient-rich plants—it emphasizes the significance of a well-balanced plant-based diet as a strategic approach to fulfill nutritional requirements. This nutritional approach not only debunks misconceptions about nutrient inadequacies but also promotes the concept that a thoughtfully chosen assortment of plant-based foods may provide athletes with a strong basis for achieving peak performance.

**Table 2:** Comparative nutrient composition in plant-based vs. non-plant-based sports diets for athletes.

Nutrient	Plant-Based Sports Diet	Non-Plant-Based Sports Diet	References
Protein	Legumes, Tofu, Tempeh, Seitan, Quinoa	Chicken, Fish, Lean Meat, Eggs	[206,207]
Omega-3 Fatty Acids	Flaxseeds, Chia Seeds, Walnuts, Algal Oil	Fatty Fish (Salmon, Mackerel), Fish Oil Supplements	[208,209]
Iron	Lentils, Chickpeas, Spinach, Pumpkin Seeds	Red Meat, Poultry, Fish, Fortified Cereals	[210,211]
Calcium	Kale, Bok Choy, Tofu (Calcium-set), Fortified Plant Milk	Dairy Products, Fortified Dairy Alternatives	[212,213]
Vitamin B12	Fortified Foods (Plant Milk, Breakfast Cereals), B12 Supplements	Animal Products (Meat, Dairy, Eggs)	[214,215]
Zinc	Lentils, Chickpeas, Pumpkin Seeds, Cashews	Meat, Shellfish, Dairy Products	[216,217]
Vitamin D	Fortified Plant Milk, Fortified Orange Juice, Sun Exposure	Fatty Fish (Salmon, Tuna), Fortified Dairy Products	[218,219]
Fiber	Whole Grains, Legumes, Nuts, Seeds, Fruits, Vegetables	Limited in Animal Products	[220,221]
Antioxidants	Berries, Dark Leafy Greens, Nuts, Seeds	Not as Prominent in Traditional Sports Diets	[222,223]
Carbohydrates	Whole Grains (Brown Rice, Quinoa), Sweet Potatoes, Fruits	Pasta, Bread, Rice (White)	[224,225]

### 4.3. Plant Compounds and Their Potential Benefits in Sports

Further investigating the connection between plant-based nutrition and athletic performance, the analysis extends to include the diverse range of bioactive chemicals naturally found in plants [226]. The aim is to explore the potential advantages of plant compounds for athletes, by shifting the attention from just the nutritional value to the complex network of phytochemicals, antioxidants, and anti-inflammatory compounds. These elements contribute to improved recovery and resilience in the field of sports. The main focus of this investigation is the examination of phytochemicals, which are plant components that provide health benefits beyond basic nutrients [227]. Athletes, in their pursuit of peak performance, may discover valuable support in these bioactive substances [228]. Phytochemicals, specifically flavonoids present in berries, citrus fruits, and tea, have been linked to anti-inflammatory and antioxidant characteristics [180,229]. Berries like strawberries and blueberries, for example, include flavonoids called anthocyanins and flavonols that have been associated with potent antioxidant effects [230]. Similarly,, flavonoids found in citrus fruits like oranges and lemons, such as hesperidin and naringenin, have anti-inflammatory properties and contribute to overall health [231]. Furthermore, catechins like epigallocatechin gallate (EGCG), which are well-known for their anti-inflammatory and antioxidant properties, are rich in tea, especially green tea [232]. This discovery broadens the range of tools available to athletes, going beyond traditional nutrition, and encouraging them to utilize the potential advantages of these substances produced from plants. Antioxidants, highly regarded for their capacity to counteract free radicals and address oxidative stress, are recognized as significant contributors in the discussion surrounding plant compounds [233]. Athletes who are facing the challenges of rigorous training might seek assistance from plant-based foods that are rich in antioxidants, such as vibrant fruits and vegetables, as well as nuts and seeds [14,234]. The study goes beyond simply listing antioxidants and delves into a detailed examination of how these substances can impact an athlete’s physiological environment, perhaps improving recovery and strengthening the body against the demands of training.

The presence of anti-inflammatory substances in plant-based diets is crucial in the field of sports nutrition [235]. The primary cause of the diet’s anti-inflammatory effects is the presence of nutrients and bioactive compounds that alter the body’s inflammatory pathways. For example, certain antioxidants found in plant-based diets, such as polyphenols and flavonoids, can prevent the production of pro-inflammatory cytokines including interleukin-6 (IL-6) and tumor necrosis factor-alpha (TNF-α) [236,237]. Furthermore, by competing with omega-6 fatty acids for enzymatic conversion into pro-inflammatory eicosanoids, omega-3 fatty acids—which are abundant in foods like flaxseeds, walnuts, and fatty fish—execute anti-inflammatory effects [238]. Additionally, eating a diet high in fiber, such as fruits, vegetables, and whole grains, encourages the growth of beneficial gut bacteria which produce short-chain fatty acids that help regulate immune responses and reduce inflammation [239]. The innate inflammatory response elicited by physical activity is an integral aspect of adaptation, however, persistent inflammation presents hazards to athletes [240]. The narrative emphasizes the presence of plant chemicals, such as curcumin in turmeric, quercetin in onions and apples, and resveratrol in grapes, which are known for their potential anti-inflammatory properties [241,242]. Athletes are encouraged to view these substances not only as supplements, but also as essential elements of a comprehensive strategy for reducing inflammation and enhancing recovery.

The narrative goes beyond the biochemical aspects to discuss the physiological consequences that athletes should expect, using specific plant chemicals as examples [243,244]. Resveratrol, which is present in red grapes, has been linked to better cardiovascular health and increased endurance [245]. The narrative presented here surpasses the reductionist perspective, encouraging athletes to not only focus on the nutritional value, but also to recognize the comprehensive advantages inherent in the wide range of plant chemicals. The focal topic of this investigation is the examination of how plant chemicals can potentially enhance recovery and resilience. The narrative invites athletes to consider plant-based nutrition as more than just a dietary choice, but as a comprehensive strategy that strengthens the body against oxidative stress, promotes anti-inflammatory responses, and enhances general resilience in the face of athletic obstacles.

## 5. Antioxidants in Plant-Based Foods

### 5.1. Overview of Antioxidants in Plants

**Figure 3:**
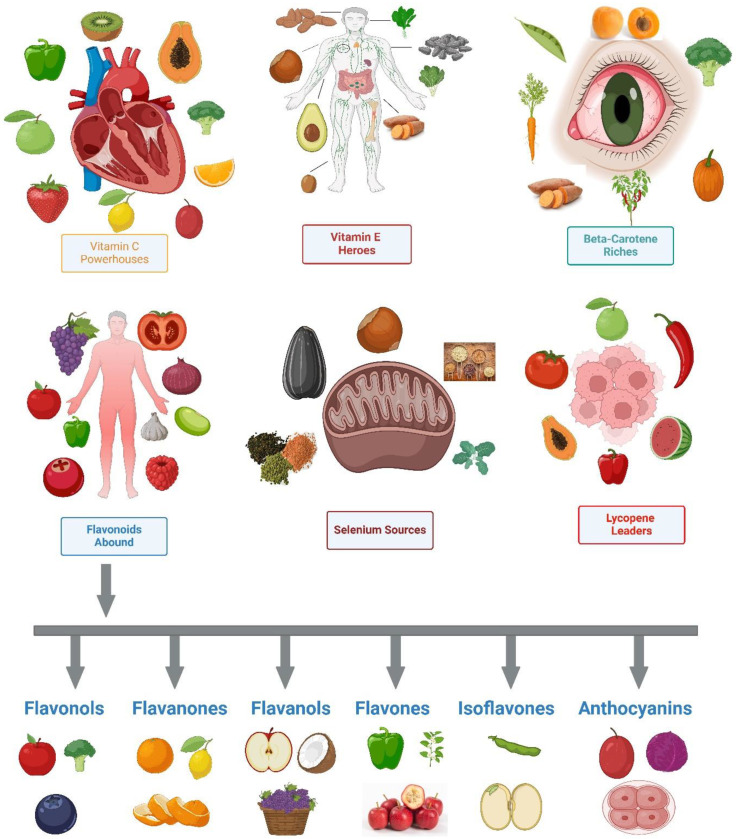
Comprehensive graphical overview of antioxidant-rich plant sources: categorization and compound identification. This figure was created with BioRender.com (accessed on 28 February 2024).

The narrative that emerges from looking into the antioxidant content of plant-based diets extends beyond the usual nutrition debates [74]. The goal is to get individuals to pay attention to the wide variety of antioxidants that plants contain. Collectively, these antioxidants enhance overall health like a symphony of defensive mechanisms (Figure 3). The examination of antioxidants in plants offers a comprehensive exploration of the diverse array of phytonutrients, each fulfilling a unique function in enhancing well-being [246]. Plants contain a variety of antioxidants, such as flavonoids in berries and carotenoids in colorful vegetables, that go beyond the usual nutrient classifications [180,247,248]. In its complex web of effects, plant-based diets involve a wide variety of chemicals, and this study casts emphasis on the idea of a single antioxidant hero.

Highlighting the wide range of antioxidants present in plants is crucial for changing the way we think about consuming antioxidants (Table 3) [249]. The conventional method frequently focuses on a limited number of well-established antioxidants, but the plant-based viewpoint expands the scope to include other lesser-known yet equally powerful antioxidants [250]. This variety encourages athletes to explore a diverse and vibrant range of plant-based foods, each providing its own combination of antioxidants to promote overall health (Figure 3).

This study explores and explains the differences in the functioning of antioxidants obtained from plant sources compared to those obtained from other dietary sources [248]. Although antioxidants obtained from supplements or animal-derived sources have a role to play, the distinct bioavailability and synergistic interactions shown in plant-based antioxidants provide a strong argument [196]. Plant-derived bioactive compounds frequently demonstrate a range of functions, such as anti-inflammatory and anti-cancer capabilities. These chemicals provide athletes with a comprehensive approach to supporting their health, which goes beyond the conventional antioxidant concept [251]. The core focus is on the synergy between various antioxidants found in plant-based meals, highlighting that their cumulative impact is more significant than the individual chemicals alone [252]. The study goes beyond simplistic viewpoints, encouraging athletes to perceive their dietary choices not as separate decisions but as contributions to a balanced symphony of health-enhancing chemicals [228,252]. This comprehensive approach encourages athletes to embrace the combined advantages inherent in entire, plant-based meals, acknowledging that the total effect is higher than the individual antioxidant components [175,178].

**Table 3:** Summary of scientific studies on sports, plant-based diets, and antioxidants: methodologies, sample sizes, and findings.

Study Title	Methodology	Sample Size	Main Findings	Reference
The Effect of Pomegranate Juice Supplementation on Strength and Soreness after Eccentric Exercise	Randomized, double-blind, placebo-controlled crossover trial	17 healthy, physically active, resistance-trained men	In resistance-trained people, twice-daily pomegranate juice supplementation lowers muscular soreness in the elbow flexor but not in the knee extensor muscles.	[253]
Effects of a single dose of beetroot juice on cycling time trial performance at ventilatory thresholds intensity in male triathletes	Randomized, double-blind, placebo-controlled crossover trial	12 well-trained, male triathletes (aged 21-47 yr)	Acute BJ supplementation does not support an improvement in the variables examined. Higher doses are needed for improving time trial performance in male triathletes during a cycle ergometer test.	[254]
Influence of tart cherry juice on indices of recovery following marathon running	Randomized, double-blind, placebo-controlled trial	20 volunteers, male (n = 13) and female (n = 7).	Demonstrated that the cherry juice reduced oxidative stress and inflammation and hence increases the rate of recovery.	[255]
Dietary antioxidant restriction affects the inflammatory response in athletes	Observational study	17 healthy endurance-trained male adults aged 18–35 years	A diet rich in carotenoids may be beneficial to combat exercise-induced oxidative stress in athletes performing exercise.	[256]
Effect of blueberry ingestion on natural killer cell counts, oxidative stress, and inflammation prior to and after 2.5 h of running	Randomized, controlled trial	25 healthy adults	Daily blueberry consumption for 6 weeks increases NK cell counts, and acute ingestion reduces oxidative stress and increases anti-inflammatory cytokines.	[257]
Effect of green tea extract supplementation on exercise-induced delayed onset muscle soreness and muscular damage	Randomized, triple-blind, placebo-controlled trial	20 healthy, untrained men	The green tea extract supplementation has positive effects on muscle recovery after strenuous exercise.	[258]
Curcumin supplementation likely attenuates delayed onset muscle soreness (DOMS)	Randomized, double-blind, controlled trial	17 healthy adults	Oral curcumin likely reduces pain associated with DOMS with some evidence for enhanced recovery of muscle performance.	[259]
A 12-Week Randomized Double-Blind Placebo-Controlled Clinical Trial, Evaluating the Effect of Supplementation with a Spinach Extract on Skeletal Muscle Fitness in Adults Older Than 50 Years of Age	Double-blind, placebo-controlled randomized trial	51 participants	In subjects, moderate-intensity strength training combined with daily supplementation for 12 weeks with a natural extract of *Spinacia oleracea* L. improved muscle-related variables and muscle quality	[260]
Effect of Exercise on Oxidative Stress: A 12-Month Randomized, Controlled Trial	12-months randomized control trial (RCT)	173 overweight women	Aerobic exercise, when accompanied by relatively marked gains in aerobic fitness, decreases oxidative stress among previously sedentary older women.	[261]
Antioxidant and anti-nociceptive effects of *Phyllanthus amarus* on improving exercise recovery in sedentary men: a randomized crossover (double-blind) design.	Randomized, double-blind, controlled trial	12 participants	Acute *Phyllanthus amarus* supplementation reduced oxidative stress and muscle soreness induced by high-intensity exercise	[262]
Consumption of an Anthocyanin-Rich Extract Made from New Zealand Blackcurrants Prior to Exercise May Assist Recovery from Oxidative Stress and Maintains Circulating Neutrophil Function: A Pilot Study	Experimental design	12 participants	Consumption of blackcurrant anthocyanin-rich extract (BAE) 1 h prior to exercise facilitated recovery from exercise-induced oxidative stress and preserved circulating neutrophil function.	[263]
A double-blind, randomized, placebo-controlled trial on the effect of Ashwagandha (*Withania somnifera* dunal.) root extract in improving cardiorespiratory endurance and recovery in healthy athletic adults	Double-blind, randomized, placebo-controlled trial	50 endurance athletes	Ashwagandha root extract can successfully enhance cardiorespiratory endurance and improve the quality of life in healthy athletic adults	[264]
Effects of Six-Week *Ginkgo biloba* Supplementation on Aerobic Performance, Blood Pro/Antioxidant Balance, and Serum Brain-Derived Neurotrophic Factor in Physically Active Men	Double-blind, placebo-controlled Trial	18 active young men	*Ginkgo biloba* extract provide improvements in endurance performance and blood antioxidant capacity, and elicit somewhat better neuroprotection through increased exercise-induced production of BDNF.	[265]
Evaluation of the Efficacy of Supplementation with Planox^®^ *Lemon verbena* Extract in Improving Oxidative Stress and Muscle Damage: A Randomized Double-Blind Controlled Trial	Randomized double-blind controlled trial	30 males and 30 females	*Lemon verbena* extract is a safe and edible natural plant extract that can reduce muscle damage and soreness after exercise.	[266]

### 5.2. Specific Antioxidants Found in Common Plant Foods

In order to comprehend the vast array of health-promoting compounds contained in staple plant foods including fruits, vegetables, grains, and legumes, it is necessary to do in-depth research on the specific antioxidants present in these meals. We delve into the complex understanding of antioxidants, their potential health benefits, and the wide range of antioxidant profiles found in different plant-based sources, going beyond just listing them. Within the domain of fruits, the array of antioxidants is extensive and varied [112]. Vitamin C, which is plentiful in citrus fruits, strawberries, and kiwi, is a powerful antioxidant that enhances the immune system [111,182]. Additionally, the anthocyanins included in berries, such as blueberries and raspberries, provide both their vivid colors and valuable properties that reduce inflammation and protect the nervous system [267,268]. In addition to common antioxidants, the narrative delves into less well-known compounds, including the apple quercetin and grape resveratrol, which each have their own unique health benefits [269,270].

Vegetables, which are essential for a diet rich in nutrients, provide a wide variety of antioxidants [181,271,272]. Carotenoids, such as beta-carotene found in carrots and sweet potatoes, lutein present in spinach, and lycopene found in tomatoes, demonstrate the wide range of antioxidants present in this food group [273,274]. These chemicals have multiple benefits, including enhancing pigmentation and playing important roles in maintaining eye health, protecting the skin, and promoting cardiovascular well-being [275]. Furthermore, cruciferous vegetables, such as broccoli and brussels sprouts, provide sulforaphane to the collection of antioxidants, which may provide anti-cancer advantages [276,277].

Grains, which are sometimes undervalued for their antioxidant content, offer a captivating variety of chemicals [278,279]. The phenolic acids present in whole grains, such as oats and brown rice, have antioxidant and anti-inflammatory properties [278,280]. Flavonoids, commonly found in fruits, also have a positive impact on grains such as buckwheat and quinoa, providing a wide range of health advantages [281,282]. This discovery challenges the traditional belief that antioxidants are only found in colored fruits and vegetables. It encourages athletes to recognize the nutritional value present in whole grains.

Legumes, which are known for their high protein content in plant-based diets, also serve as sources of antioxidants [183,283]. Soybeans include isoflavones, which have both hormonal balancing effects and antioxidant capabilities [284,285]. In addition, the polyphenols found in lentils and chickpeas enhance the antioxidant activity in legumes, providing a diverse and comprehensive approach to promoting health [286,287]. This narrative goes beyond the protein-focused perspective on legumes, urging athletes to acknowledge the comprehensive nutritional benefits of these plant-based sources of strength.

An essential aspect of our investigation involves providing detailed information about the potential health advantages linked to each antioxidant. Vitamin E, present in nuts and seeds, acts as an antioxidant and promotes skin health and immunological function [58,288]. The catechins included in green tea, which is made from the leaves of the *Camellia sinensis* plant, contribute to antioxidant and anti-inflammatory actions, potentially improving cardiovascular health [289,290]. Displaying the range of antioxidant compositions in various plant-based diets offers a visually engaging exploration of the wide spectrum of nutritional variations. The colors of berries, leafy vegetables, and turmeric represent different antioxidants, each with its own specific health benefits [1,31,190,228].

### 5.3. Synergistic Effects of Antioxidants in Plants

**Figure 4:**
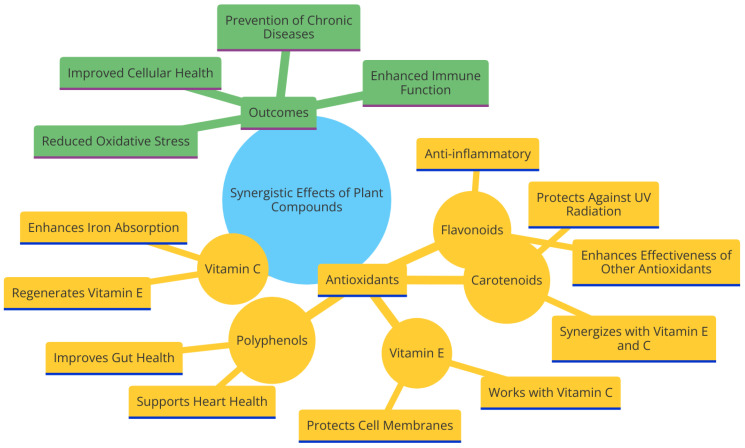
Synergistic enhancement of antioxidant efficacy through plant-derived compounds. This figure was created with Diagrams: Show Me (accessed on 13 February 2024).

To better understand the intricate relationship between various plant antioxidants and how they synergistically boost each other’s efficacy, research into plant-based nutrition has focused on the concept of antioxidant synergy (Figure 4). Beyond the conventional understanding of antioxidants in isolation, this study explores the complex interplay between plant-based diets and antioxidants, to determine the latter’s role in athletic health and performance. The idea of synergy among plant antioxidants is shown as a story that goes beyond the combined effect of each individual antioxidant [291]. The complex network of phytonutrients found in plant meals, each with its distinct antioxidant composition, work together in a coordinated manner to enhance the total antioxidant impact [292]. This interdependent interaction challenges simplistic viewpoints, encouraging athletes to perceive their food decisions not solely as suppliers of individual components, but rather as coordinated arrangements that promote good health.

The narrative is centered on explaining how antioxidants in plants can work together. Vitamin C and flavonoids, which are present in many fruits and vegetables, have a synergistic effect when taken together. A number of flavonoids have their bioavailability enhanced by vitamin C because of its tendency to increase absorption [293,294]. Beyond the obvious nutritional benefits, there is an intricate network of processes that allows athletes to get a variety of antioxidant benefits from plant-based diets. Illustrating instances of antioxidant synergy in particular plant-based meals vividly demonstrates this principle (Figure 4). When leafy greens, which are high in carotenoids, are combined with tomatoes, which are rich in vitamin C and lycopene, the resulting meal visually demonstrates the concept of antioxidant synergy [108,295]. The complex equilibrium present in this meal not only provides a wide range of antioxidants but also likely enhances their combined effect. Athletes are advised to carefully select meals that highlight this harmonious combination, acknowledging that the colors and flavors on their plate represent more than just visual appeal—they represent a deliberate strategy to enhance health and performance [296].

The vital impact of antioxidant synergy on overall health and performance is the primary foci of this study. It is recommended that athletes think about the quality as well as the quantity of antioxidants in their meals [175]. A complete diet rich in plant-based foods such fruits, vegetables, grains, and legumes is the best way to take advantage of antioxidants in their whole form [196,297]. The narrative challenges the idea that antioxidants may be easily substituted for one another and emphasizes the significance of recognizing the intricacies present in plant-based meals for achieving overall health optimization [298].

## 6. Practical Applications for Athletes

### 6.1. Incorporating Plant-Based Foods into Athletes’ Diets

Athletes need an organized strategy to incorporate plant-based nutrition into their meals [179]. The goal of these suggestions is to provide athletes with easy-to-implement instructions that they may incorporate into their dietary plans. An increase in variety, a focus on high nutritional content, and customization for different sports and training stages are the main goals. Protein should come from a variety of sources, according to the plant-based diet guidelines [178]. Diverse plant-based protein sources, such as edamame, lentils, beans, and tofu, are recommended for athletes [186]. Ensuring a comprehensive amino acid composition is the goal, as it maximizes the process of protein synthesis. Athletes are encouraged to focus on plant-based options that are minimally processed, including fruits, vegetables, whole grains, nuts, and seeds, by highlighting the importance of whole foods. Optimal nutrition consumption and improved health are the goals of this approach, which aims to reduce reliance on processed foods. It is the goal of meal planning strategies to strike a balance between nutrient density and nutritional balance. For optimal energy during training and recovery, athletes should aim to eat a balanced diet that includes carbs, proteins, and fats (the “macros”) [299]. The aesthetic value of the dish is elevated, and the variety of phytonutrients and antioxidants provided by the fruits and vegetables is assured, by using a diversified selection of these ingredients [300]. Energy levels and muscle recovery are affected by the timing of food intake, which is why this factor is given special attention when discussing exercise sessions [301]. When thinking about different sports and training times, specific dietary considerations come up [302]. Endurance athletes should fuel their lengthy training sessions with carbohydrate-rich plant foods [2]. A diet rich in iron and vitamin C is crucial for endurance athletes since their bodies need more iron than the average person [303]. On the contrary, athletes who put an emphasis on strength and power use plant-based protein sources to help with recovery and protein synthesis in the muscles. Because omega-3 fatty acids may have strong anti-inflammatory effects, they also highlight the need of eating foods high in these nutrients [304]. Nutrient intake should be structured to coincide with different training stages, and calorie consumption should be adjusted according to the quantity of training.

### 6.2. Developing Antioxidant-Rich Meal Plans for Training and Recovery

In order to help athletes in their training and recovery, this section provides customized meal plans (Table 4) that are high in antioxidants and made to fit their nutritional demands. Primarily, the content is devoted to offering sample antioxidant-rich meal plans that are designed for pre-training, post-training, and recovery phases. In addition, taking into account the different needs of athletes, the factors also include adjusting to changes based on personal dietary preferences and limitations. Recognizing the pivotal role that time and composition play in achieving optimal results, the study emphasizes their critical importance.

During the pre-training period, it is advised to follow a meal plan that focuses on providing a well-balanced combination of macronutrients to provide a consistent supply of energy during the impending physical activity [305,306]. A deliberate integration of intricate carbs, lean proteins, and nourishing fats is tactically implemented. Athletes can derive advantages from ingesting foods that are rich in antioxidants, such as berries, almonds, and leafy greens, during this phase. This helps to promote cellular health and establish a solid nutritional base for the impending activity [234,307].

Post-training meal regimens prioritize effective recovery by focusing on replenishing glycogen stores and promoting muscle regeneration [308]. The focus here is on maintaining a balanced consumption of carbs and proteins, with a significant role being played by components that are rich in antioxidants [309]. Consuming fruits that are rich in vitamin C, such as citrus fruits or kiwi, might help decrease oxidative stress [310,311]. Additionally, including plant-based protein sources like beans or tofu in diet guarantees a well-rounded dietary profile that supports recovery [312].

The recovery phase highlights the need of restoring energy reserves and promoting muscle restoration [313]. The meal plan suggests incorporating a blend of carbohydrates and proteins, while also emphasizing the use of anti-inflammatory foods [314,315]. Adding vibrant vegetables such as bell peppers and tomatoes, which are recognized for their antioxidant capabilities, is crucial in reducing inflammation caused by exercise [316]. Customizing these regimens to suit individual nutritional preferences guarantees that athletes may follow the guidelines while meeting their distinct likes and limitations.

Within these suggestions, the study continuously highlights the crucial elements of timing and composition. Consuming these meals rich in antioxidants at the appropriate time corresponds to the distinct requirements of each phase, be it preparing the body for physical activity, aiding in recuperation, or enhancing cellular health during periods of rest [309]. The meal plans are carefully designed to incorporate a balanced combination of macronutrients and micronutrients, acknowledging that the interaction between these components enhances the overall efficacy of the dietary approach [317].

**Table 4:** Recommended antioxidant-rich plant-based foods for athletes: a concise guide.

Food Group	Food Options	Key Antioxidants	Benefits for Athletes	Additional Nutrients	Reference
Protein Powerhouses	Lentils	Phenolic acids, Flavonoids	Muscle repair, satiety, sustained energy	Fiber, Iron, Folate	[194,318]
	Tempeh	Isoflavones, Lunasin	Muscle building, immune function	Iron, Calcium, Prebiotics	[319,320]
	Quinoa	Quercetin, Kaempferol	Reduced inflammation, improved recovery	Fiber, Magnesium, Iron	[224,321]
	Tofu	Genistein, Daidzein	Bone health, muscle preservation	Calcium, Iron, Manganese	[322,323]
	Nuts and Seeds (almonds, chia, hemp)	Vitamin E, Selenium	Reduced oxidative stress, cell protection	Healthy fats, Fiber, Minerals	[324,325]
Fuel for Performance (high in complex carbs)	Brown rice	Anthocyanins, Phenolic acids	Sustained energy release, blood sugar control	Fiber, Manganese, B vitamins	[326,327]
	Sweet potatoes	Beta-carotene, Chlorogenic acid	Improved blood flow, muscle endurance	Vitamin A, Potassium, Fiber	[328,329]
	Oats	Avenanthramides, Ferulic acid	Reduced inflammation, improved recovery	Fiber, beta-glucan, Magnesium	[330,331]
Vitamin & Mineral Champions	Berries (blueberries, strawberries)	Anthocyanins, Ellagic acid	Improved cognitive function, reduced muscle soreness	Vitamin C, Fiber, Potassium	[332,333,334]
	Leafy greens (kale, spinach)	Vitamin C, Lutein	Bone health, immune function, eye health	Vitamin K, Folate, Calcium	[335,336]
	Cruciferous vegetables (broccoli, cauliflower)	Glucosinolates, Sulforaphane	Detoxification, cancer prevention	Fiber, Vitamin C, Potassium	[337,338]
	Fortified plant milks (calcium, vitamin D)	Calcium, Vitamin D	Bone health, immune function	Vitamin B12, Riboflavin	[339,340]
Omega-3 Superstars	Chia seeds	Alpha-linolenic acid (ALA)	Brain health, reduced inflammation	Fiber, Protein, Calcium	[341,342]
	Algae oil (DHA/EPA supplements)	Docosahexaenoic acid (DHA), Eicosapentaenoic acid (EPA)	Cognitive function, muscle recovery, anti-inflammatory properties	Vitamin E	[343,344]
Natural healer	Turmeric	Curcumin	Anti-inflammatory properties	Flavonoids	[345,346]

### 6.3. Considerations for Different Types of Sports

The primary objective is to address the unique needs of various sports disciplines by offering specialized insights and concerns regarding plant-based nutrition. Realizing that different types of athletes have different nutritional needs and challenges, this section delves into the nuances of plant-based nutrition for strength athletes, endurance athletes, and team sports participants. Table 5 demonstrates the instructions on how to include plant-based nourishment in different types of sports. Endurance athletes, who engage in lengthy periods of sustained effort during training and competition, need to focus on fueling methods that prioritize plant-based diets rich in carbohydrates [179]. The meal plan may include whole grains, fruits, and vegetables to ensure a consistent and sustained release of energy [347]. To cater to the distinct dietary requirements of endurance athletes, it is important to focus on obtaining adequate amounts of iron and vitamin C, the nutrients that are crucial for meeting the increased demands of endurance exercise, especially in situations when iron absorption may be affected [303].

Strength athletes, whose focus are muscular development and strength, derive advantages from a plant-based nutrition strategy that emphasizes protein-rich sources [178]. Suggestions may involve incorporating plant-based proteins such as lentils, tofu, and seitan to enhance muscle protein synthesis [358,359]. Furthermore, incorporating foods that are abundant in omega-3 fatty acids, such as flaxseeds and chia seeds, can be beneficial due to their possible anti-inflammatory properties, which aid in the process of healing and promote general muscular well-being [360,361]. Team sports pose distinctive challenges and requirements, necessitating athletes to participate in sporadic episodes of vigorous exertion [362]. Plant-based nutrition considerations for team sports entail the delicate equilibrium between energy demands and the need for rapid bursts of strength and agility [14,363]. It is essential to create meal plans that include a combination of carbohydrates, proteins, and nutritious fats [364]. To address the requirement for quick recuperation between periods of physical activity, it may be necessary to strategically consume foods that are rich in antioxidants in order to alleviate oxidative stress [365,366].

Given the acknowledgement that a single approach is not suitable for everyone, it becomes crucial to provide practical guidance on how to adjust plant-based nutrition to various sports situations [367]. Athletes are advised to explore various plant-based sources, modify macronutrient ratios according to different training phases, and take into account individual preferences and tolerances [189]. The adaptability of the guidelines guarantees that they are in line with the specific requirements of each sport and the distinct physiological reactions of individual athletes [367].

## 7. Challenges and Limitations

Examining the nuances of plant-based diets in sports reveals significant benefits as well as challenges for athletes considering adopting this nutritional approach. The primary areas of concern for athletes aiming for peak performance—maintaining energy balance, protein consumption, and dietary deficiencies—are discussed in relation to the possible downsides. Because of misconceptions regarding its availability in plant sources, protein—which is frequently emphasized for its vital role in muscle growth and recovery—poses a problem to those following plant-based diets. However, research shows that when included in a diversified and well-planned diet, plant-based proteins like those found in beans, quinoa, and soy products are sufficient to meet the protein requirements of athletes. Another issue covered in the study is nutritional deficits, specifically in the areas of vitamins B12, iron, calcium, and omega-3 fatty acids. It recommends a varied plant-based diet and fortified meals and supplements as practical ways to make sure athletes are not compromising on vital nutrients. Achieving the right energy intake is also essential, particularly in light of the high calorie requirements of sports training and competition. Incorporating foods high in energy from plants and carefully planning meal compositions to support nutrient balance and energy requirements are two strategies for overcoming these challenges.

**Figure 5:**
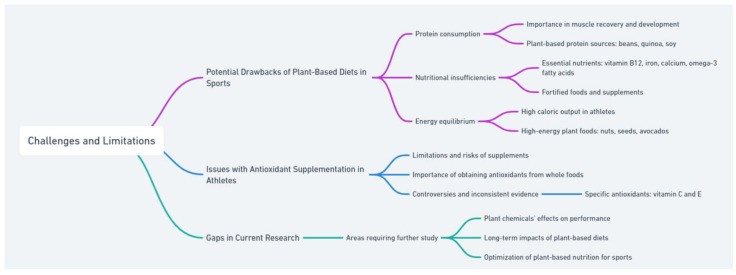
Challenges and limitations of plant-based diets and antioxidant supplementation in sports. This figure was created with Whimsical.com (accessed on 22 February 2024).

Challenges with antioxidant supplements in sports nutrition are also explored in the story (Figure 5). Although antioxidants play a crucial role in preventing oxidative stress caused by strenuous exercise, there are risks associated with using supplements rather than obtaining them from whole foods, such as overconsumption and nutritional imbalances. A diet rich in fruits, vegetables, nuts, and seeds is the most effective way to absorb antioxidants, according to the report, because they work synergistically and are more bioavailable. In addition to addressing the limitations and potential risks of supplement reliance, this approach promotes the idea that athletes should prioritize the complexity of dietary matrices above the use of isolated nutritional supplements. Although the study highlights the value of whole foods in providing a balanced array of antioxidants and other micronutrients necessary for optimal health and athletic performance, the ongoing debate over the effectiveness of antioxidant supplements in improving performance and recovery adds another layer of complexity to the matter.

The discussion emphasizes the need for more scientific studies to address current gaps in the field of sports nutrition, plant-based diets, and antioxidants. Although progress has been made in recognizing the advantages of plant-based nutrition for athletes, there are still unexplored areas, such as the specific mechanisms by which plant chemicals affect performance and the lasting impacts of these diets in various sports. The narrative suggests that future study should investigate the precise effects of phytochemicals and antioxidants found in plant-based meals on athletic performance and recovery. Future research should prioritize customized nutritional approaches that take into account the varied requirements of different sports, training levels, and individual athlete needs. The goal is to improve plant-based nutrition strategies to help athletes reach their performance goals and preserve their health and well-being. In sum, plant-based diets can be a beneficial nutritional approach for athletes, but it is crucial to address the problems related to protein consumption, vitamin shortages, and energy equilibrium. Prioritizing whole foods for antioxidants and advocating for more research to address knowledge gaps highlights a sophisticated approach to sports nutrition that focuses on evidence-based dietary planning.

## 8. Future Directions and Research Opportunities

**Figure 6:**
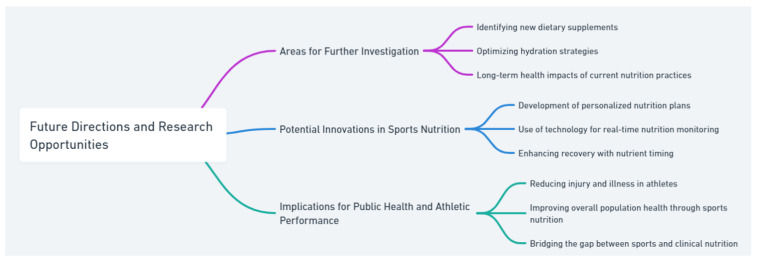
Charting the future: directions and opportunities in sports nutrition research. This figure was created with Whimsical.com (accessed on 22 February 2024).

The future of sports nutrition is found in the intersection of athletics, plant-based diets, and antioxidants (Figure 6). This junction requires more research to understand the intricate connections and possible advantages for athletic performance and public health. This investigation requires a multidisciplinary strategy that incorporates new technologies such as metabolomics and nutrigenomics to customize nutrition, recognizing individual differences in how they respond to plant chemicals. This research intends to enhance athletes’ performance with personalized dietary methods and also has the potential to reduce chronic health problems in the general population by promoting plant-based, antioxidant-rich diets. Advancements in food technology, such as precise fermentation and novel plant-based protein sources, are expected to transform sports nutrition by improving nutrient absorption and recovery mechanisms, ultimately promoting muscle health and overall well-being. Personalized nutrition plans utilizing advanced research and technology provide specific dietary advice tailored to individual athletes, maximizing the synergy of sports, plants, and antioxidants. Personalized nutrition, coupled with advanced technologies, might offer practical, data-based advice for improving diets, marking the beginning of a new phase in sports nutrition that is tailored and based on scientific research.

Beyond athletic performance, the shift towards plant-based nutrition has important public health implications since it has the potential to reduce chronic diseases and enhance overall well-being. This change necessitates a focused approach in education and policy development to promote healthy eating habits among the general public, emphasizing the importance of antioxidants and plant-based diets in enhancing quality of life and lowering healthcare costs. Collaboration among researchers, policymakers, and educators to promote these nutritional strategies could lead to a societal shift towards healthier lifestyles, emphasizing the crucial role of nutrition in sports performance, public health, and overall quality of life (Figure 6).

## 9. Conclusions

**Figure 7:**
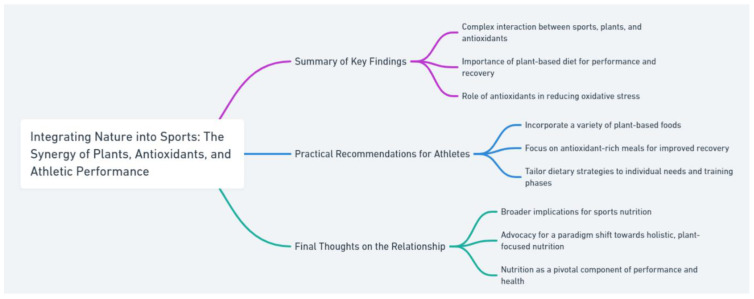
Integrating nature into sports: the synergy of plants, antioxidants, and athletic performance. This figure was created with Whimsical.com (accessed on 22 February 2024).

The present study explores the complicated interactions among sports, plant-based diets, and antioxidants, revealing how they affect athletic performance and recovery. We have shown that plant-based diet plays a crucial role in improving athletic performance, especially by utilizing antioxidants to reduce oxidative stress. The complex interaction among different plant compounds and the personalized potential of dietary approaches were highlighted as significant subjects, expanding our knowledge of sports nutrition. Research on oxidative stress has shown that it can serve as a stimulus for adaptability and also as a possible cause of harm, providing a balanced view of the issues athletes encounter. The increasing popularity and acknowledged advantages of plant-based diets in sports, as well as the challenges of implementing such dietary regimens, have shed light on the complicated connection between sports nutrition and plant-based diets (Figure 7).

Based on the gained insights, athletes should prioritize including antioxidant-rich plant-based foods into their diets to promote cellular health and improve recovery. The recommendations emphasize the importance of personalized dietary strategies tailored to each athlete’s individual physiological characteristics and nutritional requirements, advocating for dietary customization to maximize the benefits of plant-based nutrition and antioxidants in sports. This investigation emphasizes the transforming power of comprehending the interaction among sports, plants, and antioxidants. It functions as both an academic study and a useful manual for athletes, researchers, and professionals dealing with the changing field of sports nutrition. This change in perspective encourages a reassessment of the importance of nutrition in athletic performance and health, indicating a trend towards a more comprehensive, plant-focused, and personalized approach to sports nutrition. This evolution demonstrates how food choices are evolving and emphasizes nutrition as a dynamic force that can greatly improve physical performance in different fields. This review summarizes the current knowledge and suggests a direction for future study and practical use, representing a significant moment in the development of sports nutrition. All stakeholders are encouraged to engage in this ongoing discussion about the intersection of sports, plants, and antioxidants, which aims to serve as a comprehensive guide for athletes seeking to enhance their performance, recovery, and overall well-being.

## Figures and Tables

**Table 1:** A comprehensive compilation of distinct antioxidants in commonly consumed plant-based food.

Plant Food Category	Specific Plant Foods	Antioxidants Present	References
Fruits	Berries (Blueberries, Strawberries, Raspberries), Citrus fruits (Oranges, Grapefruits, Lemons), Apples, Pears, Cherries	Anthocyanins, Vitamin C, Quercetin, Flavonoids	[45,46,47]
Vegetables	Leafy greens (Spinach, Kale, Swiss Chard), Cruciferous vegetables (Broccoli, Cauliflower), Bell peppers, Tomatoes, Carrots	Quercetin, Polyphenols, Lutein, Zeaxanthin, Vitamin E, Glucosinolates, Sulforaphane, Indoles, Vitamin C, Vitamin A, Lycopene, Beta-carotene,	[48,49]
Nuts and Seeds	Almonds, Walnuts, Chia Seeds, Flaxseeds, Sunflower seeds, Pumpkin seeds	Vitamin E, Omega-3 fatty acids, Polyphenols, Selenium, Vitamin E	[50,51]
Legumes	Chickpeas, Lentils, Black beans, Peanuts, Pinto beans, Kidney beans	Flavonoids, Resveratrol, Coenzyme Q10, Isoflavones	[52,53]
Whole Grains	Quinoa, Brown rice, Oats, Barley, Whole wheat	Vitamin E, Selenium, Polyphenols, Ferulic Acid, Beta-glucans	[54,55]

**Table 5:** Practical guidelines for optimal antioxidant intake through plant-based foods in sports nutrition.

Food Group	Antioxidant-Rich Examples	Serving Recommendations	Benefits	References
Fruits (5 servings per day)	Berries (blueberries, strawberries, raspberries), citrus fruits (oranges, grapefruits, kiwis), pomegranates, pineapple, mangoes, apples, pears	1–2 servings per meal, snack on fruits in between	Rich in vitamin C, flavonoids, anthocyanins; boost immunity, reduce inflammation, protect against muscle damage	[348,349]
Vegetables (5 servings per day)	Cruciferous vegetables (broccoli, kale, Brussels sprouts), leafy greens (spinach, swiss chard, collard greens), bell peppers, sweet potatoes, carrots, onions, tomatoes	1–2 servings per meal, incorporate vegetables into snacks	Abundant in carotenoids, vitamin C, and other phytonutrients; enhance cell health, improve antioxidant defenses, support recovery	[348,350]
Legumes (2–3 servings per week)	Lentils, black beans, chickpeas, kidney beans, soybeans	1/2 cup cooked legumes per meal, incorporate into salads, soups, and dips	Excellent source of plant-based protein, fiber, and various antioxidants; contribute to muscle building, satiety, and overall wellbeing	[351,352]
Nuts and seeds (1–2 servings per day)	Walnuts, almonds, chia seeds, flaxseeds, hemp seeds	1/4 cup nuts or 2 tablespoons seeds per day, sprinkle on yogurt, salads, or add to smoothies	High in polyunsaturated fats, vitamin E, and minerals; provide sustained energy, promote cell health, aid in recovery	[351,353]
Whole grains (3–5 servings per day)	Quinoa, brown rice, whole-wheat bread, oats, barley	1/2 cup cooked grains per meal, choose whole-grain bread and cereals	Rich in fiber, vitamin E, and B vitamins; support gut health, regulate blood sugar, improve energy levels	[354,355]
Spices and herbs (daily)	Turmeric, ginger, garlic, cinnamon, parsley, rosemary, oregano	Add to cooking, sprinkle on meals, use in teas and infusions	Contain powerful antioxidants and anti-inflammatory compounds; enhance flavor, boost digestion, and provide additional health benefits	[356,357]
